# Association of circulating gene expression signatures with stiffness following total knee arthroplasty for osteoarthritis: a pilot study

**DOI:** 10.1038/s41598-022-16868-y

**Published:** 2022-07-25

**Authors:** Meghan A. Kirksey, Samantha G. Lessard, Marjan Khan, George A. Birch, David Oliver, Purva Singh, Valeria Rotundo, Alexandra Sideris, TKAF Consortium, Tania Pannellini, Allina A. Nocon, Mark Youseff, Paul Guirguis, Thomas W. Bauer, Eric A. Bogner, Mathias P. Bostrom, Steven B. Haas, Kethy M. Jules-Elysee, Mark P. Figgie, David J. Mayman, Alexander S. McLawhorn, Michael B. Cross, Douglas E. Padgett, Alessandra B. Pernis, Scott A. Rodeo, Kathleen Tam, Geoffrey H. Westrich, Hollis G. Potter, Matthew F. Koff, Lionel B. Ivashkiv, Thomas P. Sculco, Timothy M. Wright, Alejandro Gonzalez Della Valle, Michael L. Parks, Peter K. Sculco, Miguel Otero

**Affiliations:** 1 Hospital for Special Surgery, New York, NY 10021 USA; 2 Department of Anesthesiology, Critical Care, and Pain Management, Hospital for Special Surgery, New York, NY 10021 USA; 3 Weill Cornell Medical College, New York, NY 10021 USA; 4 HSS Research Institute, Hospital for Special Surgery, New York, NY 10021 USA; 5 Orthopedic Soft Tissue Research Program, Hospital for Special Surgery, New York, NY 10021 USA; 6 The David Z. Rosensweig Genomics Research Center, Hospital for Special Surgery, New York, NY 10021 USA; 7 The Stavros Niarchos Foundation Complex Joint Reconstruction Center, Hospital for Special Surgery, New York, NY 10021 USA

**Keywords:** Osteoarthritis, Surgery, Outcomes research, Transcriptomics

## Abstract

A subset of patients undergoing total knee arthroplasty (TKA) for knee osteoarthritis develop debilitating knee stiffness (reduced range of motion) for poorly understood reasons. Dysregulated inflammatory and immune responses to surgery correlate with reduced surgical outcomes, but the dysregulated gene signatures in patients with stiffness after TKA are poorly defined. As a consequence, we are limited in our ability to identify patients at risk of developing poor surgical outcomes and develop preventative approaches. In this pilot study we aimed to identify perioperative blood gene signatures in patients undergoing TKA for knee osteoarthritis and its association with early surgical outcomes, specifically knee range of motion. To do this, we integrated clinical outcomes collected at 6 weeks after surgery with transcriptomics analyses in blood samples collected immediately before surgery and at 24 h after surgery. We found that patients with stiffness at 6 weeks after surgery have a more variable and attenuated circulating gene expression response immediately after surgery. Our results suggest that patients with stiffness following TKA may have distinct gene expression signatures detectable in peripheral blood in the immediate postoperative period.

## Introduction

Osteoarthritis (OA) is a multifactorial disorder affecting all joint tissues and involving biomechanical and biochemical factors and maladaptive repair responses^1^. OA is in part an inflammatory condition^2^, and dysregulated local and peripheral immune/inflammatory signatures are associated with symptoms and disease activity^3^ that can predict outcomes after surgery^4^. The complex pathogenesis of OA represent significant challenges for the development of efficacious non-surgical therapeutic strategies. Currently few viable alternatives to total joint arthroplasty (TJA) exist for the treatment of end-stage OA.

TJA, most commonly of the hip or the knee (TKA), is one of the most successful and common orthopedic procedures. Over a million joint replacement surgeries are performed annually in the United States, and these numbers are projected to grow several-fold^5,6^. In spite of high overall success rates, approximately 20% of patients undergoing TKA report suboptimal outcomes. In some cases, complications occur within months after surgery^7^.

Knee stiffness, defined as limited range-of-motion that affects daily activities, occurs in 1–16% patients after TKA^8–11^. Knee stiffness results from the interplay between patient-intrinsic predisposing (pre-operative) risk factors and intra-operative and post-operative factors^12^. However, the disease- and surgery-specific signatures dysregulated in patients who develop stiffness after TKA are not well understood. As a consequence, we are limited in our ability to identify OA patients at risk of developing stiffness after TKA and to obtain the mechanistic understanding necessary to develop preventative therapies.

Using whole blood samples collected within 72 h after surgery, Gaudillière et al. identified correlations between signaling responses in CD14+ monocyte subsets with recovery from hip surgery^13^. Microarray analyses in blood samples collected before and after surgery identified changes in gene expression associated with the development of persistent pain after TKA^14^. Using serum and synovial fluid samples from patients undergoing TKA, we recently reported a distinct cytokine profile in patients who developed stiffness early after TKA^9^, and we were able to correlate perioperative cytokine levels with persistent postoperative pain after TKA^15^. Together, these results indicate that perioperative circulating gene signatures may be useful prognostic markers to identify patients at risk of developing complications following surgery.

In this pilot study using our well-characterized prospective patient cohort^9,15^, we examined the response to TKA in peripheral blood mononuclear cells (PBMCs) and whole blood, and explored the hypothesis that perioperative gene expression signatures associated with stiffness at 6 weeks after TKA can be identified in whole blood. To do this, we integrated RNA-seq analyses from PAXgene blood RNA and NanoString analyses of RNA from PBMCs, aiming to use comprehensive and unbiased transcriptomics analyses to identify general early circulating gene expression responses to surgery. Using clinical outcomes, we identified patients with stiffness after surgery and matched control patients, and we performed RNA-seq analyses of RNA from PAXgene blood RNA tubes collected on the day of surgery and at 24 h after surgery to identify changes in gene expression associated with stiffness.

## Patients and methods

### Patients and selection of controls and cases

**Figure 1:**
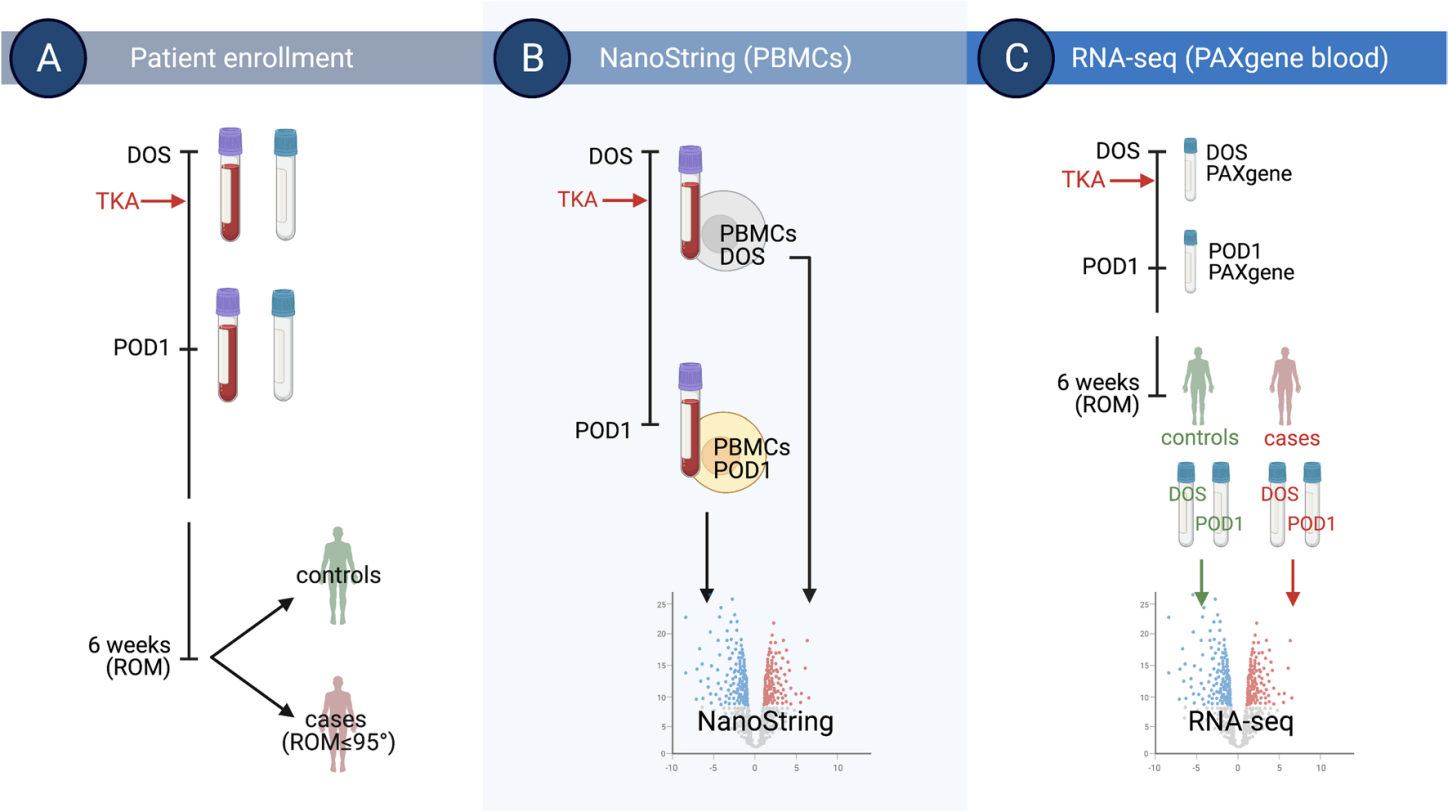
Study workflow. (**A**) We collected peripheral blood mononuclear cells (PBMCs) and PAXgene blood RNA tubes the day of surgery (DOS) and at 24 h after surgery (POD1) in patients with knee osteoarthritis (OA) undergoing total knee arthroplasty (TKA). At 6 weeks after surgery, patients were classified as cases and controls based on range-of-motion (ROM). Stiffness was defined as ROM ≤ 95° measured by goniometer. (**B**) RNA isolated from PBMCs was used for NanoString gene expression analyses to compare POD1 and DOS gene expression profiles. (**C**) RNA isolated from PAXgene blood RNA tubes was used for RNA-seq, comparing POD1 vs. DOS samples from controls and cases. Created with BioRender.com.

The study protocol was approved by the Institutional Review Board (IRB) of the Hospital for Special Surgery (HSS). Written informed consent was obtained from all participants before entering the study, and the study and all methods were performed in accordance with the relevant guidelines and regulations. After IRB approval (IRB#2015–361) and patient consent, we prospectively enrolled a cohort of 179 patients with idiopathic end-stage OA scheduled for TKA. For specific details of this patient cohort, please see references^9,15^. For this case–control level III evidence pilot study, we used samples from cases and matched controls identified at enrollment end, retrieved from surgeries performed at a high-volume orthopedic hospital by 7 expert Fellowship trained arthroplasty surgeons. Patients were enrolled by participating surgeons from a single academic institution between May 2016 and February 2018. The trial was registered before patient enrollment at clinicaltrials.gov (NCT02626533). Data were collected and hosted electronically through the Weill Cornell Medicine (WCM) Clinical and Translational Science Center (CTSC) Research Electronic Data Capture (REDCap). As previously reported, 17 patients were excluded from analysis after enrollment, leaving 162 patients for analysis of clinical data and biological samples^9,15^. PBMCs were isolated for NanoString analyses from a subset of 6 consecutive patients at the start of enrollment. PAXgene Blood RNA tubes were collected from all enrolled subjects, and samples for RNA-seq analyses were selected from patients who develop stiffness at 6 weeks after surgery and from matched controls. Patients were divided into 2 groups based on range of motion: (a) cases: patients who were found to have postoperative stiffness at 6 weeks, and (b) matched controls: patients without post-TKA stiffness at 6 weeks. Stiffness was defined as range-of-motion ≤ 95° measured by goniometer at 6 weeks (± 2 weeks)^11^, as described^9^. Cases and controls were matched by sex, race, BMI (± 4 kg/m^2^), and age (± 10 years). See the study workflow in Fig. 1.

### Peripheral blood mononuclear cells (PBMCs)

PBMCs were isolated from six consecutive patients, using heparinized venous blood samples (10–20 ml) retrieved by hospital phlebotomists immediately before surgery (day-of-surgery, DOS) and at 24 h after surgery (post-operative day 1, POD1). Whole blood was processed within 2 h of extraction by centrifugation, using Ficoll-Paque PLUS (GE Healthcare) density gradient according to manufacturer’s instructions. Isolated PBMCs were pelleted and re-suspended in RLT Buffer (Qiagen) containing 1% 2-Mercaptoethanol for RNA isolation and NanoString analyses.

### PAXgene blood RNA tubes

PAXgene Blood RNA tubes (BD Bioscences catalog # 762165) were obtained from each study participant by hospital phlebotomists at DOS and POD1 and processed according to the manufacturer’s instructions. Briefly, immediately after specimen collection the tubes were transported to the laboratory, kept at room temperature (18–25 °C) for 2 h, transferred to a − 20 °C freezer for 24 h, and stored at − 80 °C until processing for RNA isolation and RNA-seq analyses.

### RNA isolation

RNA was isolated from PBMCs using the RNeasy mini kit (Qiagen, catalog # 74104) following manufacturer’s instructions. Briefly, PBMCs were homogenized in RLT Buffer with 1% 2-Mercaptoethanol, and RNA was isolated using the Rneasy kit. RNA isolation and globin depletion from the PAXgene Blood RNA tubes were conducted at the Core Laboratory of the WCM CTSC. Briefly, RNA was isolated using the PAXgene Blood RNA kit (Qiagen catalog # 762164) following manufacturer’s instructions. After RNA isolation, a globin depletion step was performed in all samples using the GLOBINclear kit (ThermoFisher, catalog # AM1980), following the manufacturer’s instructions. RNA integrity was assessed at the WCM Epigenomics Core Laboratories Center for all samples. Only total RNA samples with sufficient concentration and with RIN > 7 and 260/280 > 1.8 were used for NanoString and RNA-seq analyses.

### nCounter NanoString gene expression analyses

For NanoString gene expression analyses, we used 100 ng of total RNA (RIN > 8, 260/280 > 1.8) isolated from PBMCs isolated from whole blood retrieved from 6 patients undergoing TKA (12 samples, 6 DOS and 6 POD1). Analyses were performed using the nCounter Immunology Pathways Panel (NanoString Technologies, Seattle, WA), following manufacturer’s instructions. Data analysis was performed using the accompanying software (nSolver 4.0, NanoString Technologies, Seattle, WA).

### RNA sequencing (RNA-seq)

After RNA isolation and globin depletion, a total of 36 RNA samples (RIN > 7, 260/280 > 1.8) isolated from PAXgene tubes collected at DOS and POD1 from 18 patients (8 cases and 10 matched controls) passed the RNA integrity quality controls and were used for RNA-seq, following standard procedures. A total of 100 ng of RNA were used to construct libraries, and sequencing was performed using an Illumina HiSeq 2500 at the WCM Epigenomics Core Facility using standard protocols^16^.

### Bioinformatics analyses

After sequencing, the reads were processed using established pipelines at the David Z. Rosensweig Genomics Research Center at Hospital for Special Surgery (HSS). Briefly, reads were pre-processed using fastp^17^, which supports adapter trimming, low quality base trimming, and calculation of additional QC metrics. Trimmed, high quality reads were aligned to the target genome using STAR^18^. Low quality and multimapping alignments were filtered out using SAMtools^19^. Reads were counted within exons and summarized at the gene level using featureCounts^20^ to produce a count matrix of reads-per-gene. These counts were analyzed for differential expression using the edgeR QLF framework^21^. Differentially expressed genes were used to perform QuSAGE pathway analyses^22^ against MsigDB^23^ pathways and gene sets. Transcription factor regulatory networks were generated from AnimalTFDB 3.0^24^ and RegNetwork^25^, which include both transcription factors and transcriptional co-factors. All results were visualized with plotly^26^ on an R Shiny^27^ platform developed by the HSS Genomics Center.

### Statistical analyses

Analyses of demographics and range-of-motion at baseline and 6 weeks after TKA were conducted by the Department of Anesthesiology, Critical Care & Pain Management at HSS to identify patients with knee stiffness (cases) and matched non-stiff patients (controls). Samples were identified after enrollment end. Unpaired Student *t*-test was used to establish statistical significance between DOS and POD1 pathway and cell scores using GraphPad Prism 8 Software (GraphPad Software, San Diego, CA).

## Results

### NanoString analyses identified surgery-induced gene expression signatures in PBMCs

To establish circulating gene expression responses to TKA, first we analyzed RNA from PBMCs collected from 6 patients at DOS and POD1 using the human nCounter NanoString Immunology panel. This panel contains 594 genes grouped in different gene sets and permits the multiplexed evaluation of changes in gene expression associated with immune cell types and functional pathways. The initial assessment identified gene expression changes consistent with responses to surgery in all patients except one (Supplementary Figure S1). Chart review revealed that this patient received twice the dose of two anti-inflammatory medications (ketorolac and meloxicam) relative to the other patients. Thus, this patient’s samples were excluded from the final analyses.

**Figure 2:**
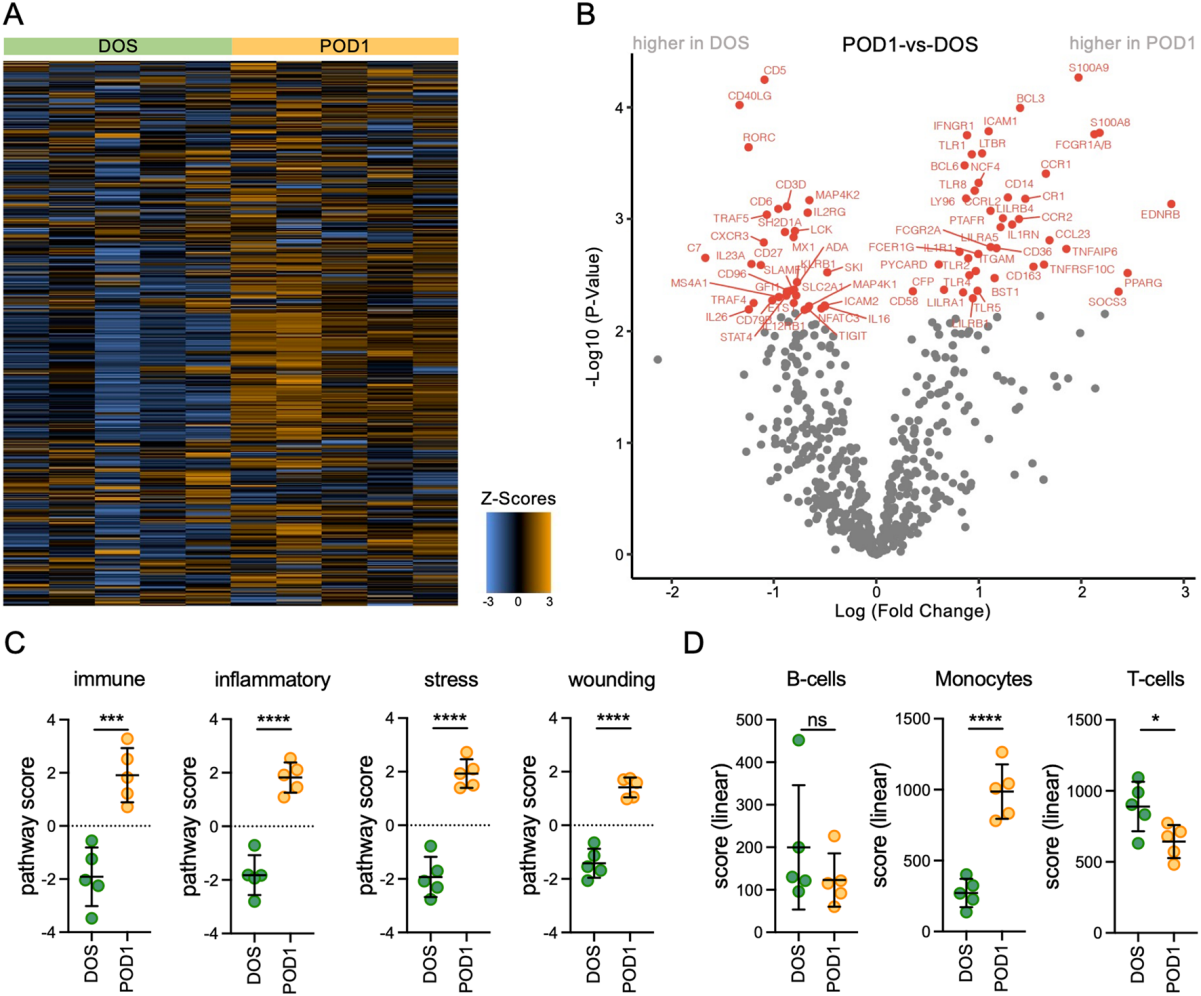
NanoString gene expression analyses of the responses to surgery of peripheral blood mononuclear cells. (**A**) Heatmap representation generated via unsupervised clustering of the normalized gene expression analyses from RNA isolated from peripheral blood mononuclear cells (PBMCs) obtained the day of surgery (DOS) and at 24 h after surgery (POD1) from 5 patients undergoing total knee arthroplasty (TKA) for knee osteoarthritis (OA). Each column represents data from samples collected from one patient on the DOS or at POD1. Each row represents relative gene expression. Orange indicates high expression, and blue indicates low expression. Data were Z-score normalized, scaled to give all genes equal variance. (**B**) Volcano plot representation of the differentially expressed genes (red, *p* < 0.05) comparing POD1 to DOS. (**C**) Representation of the pathway scores for the immune response, inflammation response, response to stress, and response to wounding in PBMCs isolated from DOS and POD1 whole blood. (**D**) Representation of the linear scores for B-cells, monocytes, and T-cells in PBMCs isolated from DOS and POD1 whole blood. Data are represented as means ± S.D. (error bars). Graphs were created with GraphPad Prism 8 (GraphPad Software, San Diego, CA). *p < 0.05 **p < 0.001, ***p < 0.0001 and ****p < 0.00001 by t-test. ns indicates not significant.

NanoString analyses showed significant gene expression changes at 24 h after surgery as shown in the heat map representation in Fig. 2A. Differential gene expression analyses identified changes in S100A8, S100A9, SOCS3, CD163, CCR1, CCR2, CR1, BLC3, STAT3, and CXCL1 mRNA, as shown in the volcano plot representation in Fig. 2B. The top 20 upregulated genes (log2FC > 1.5, *p* < 0.05) on POD1 are shown in Table 1. We next used the differentially expressed genes (DEGs) identified by NanoString to perform pathway and cell score analyses. These analyses uncovered a relative enrichment in the immune response genes (including significant changes in XBP1, NCF4, GPI, BST1, or CEBPB mRNA; adjusted *p* value < 0.05), inflammatory response genes (with significant changes in S100A9, S100A8, CCR1, PTAFR, or CYBB; adjusted *p* value < 0.05), response to stress genes (with significant changes in S100A9, S100A8, PTAFR, MAPK14, or CYBB; adjusted *p* value < 0.05), and response to wounding genes (driven by increased expression of S100A9, S100A8 and CYBB; adjusted *p* value < 0.05) at POD1 (Fig. 2C). We also observed a relative enrichment in monocyte cell scores (driven by increased CD136 mRNA) accompanied by decreased T-cell scores (determined by changes in the expression of CD6, SH2D1A, CD3E and CD3D mRNA) at POD1 (Fig. 2D), consistent with reports showing increased monocytes and decreased T cells early after surgery^13^. See Supplementary Table S1 for the NanoString normalized counts and Supplementary Table S2 for the NanoString pathway and cell-score analyses.

**Table 1:** Top 20 differentially expressed genes identified by NanoString analyses in PBMCs obtained the day-of-surgery (DOS) and at 24 h after surgery (POD1) from 5 patients undergoing total knee arthroplasty for knee osteoarthritis, with Log2 fold-change > 1.5 and *p* < 0.05.

Gene name	Log2FC	Lower	Upper	*p* value
*CXCL1*	2.59	1.57	3.61	0.00109
*S100A8*	2.53	2.1	2.96	2.99E−06
*SOCS3*	2.45	1.59	3.32	0.000531
*S100A9*	2.34	1.99	2.69	1.07E−06
*CXCR1*	2.29	1.56	3.03	0.000288
*TNFRSF10C*	2.13	1.61	2.66	4.60E−05
*EGR1*	2.04	1.22	2.85	0.0012
*CCR1*	2.01	1.53	2.49	3.74E−05
*CR1*	1.89	1.55	2.22	4.01E−06
*CD163*	1.88	1.38	2.38	7.89E−05
*CCR2*	1.8	1.29	2.31	0.000123
*BCL3*	1.78	1.44	2.12	7.30E−06
*IL1RN*	1.69	1.31	2.07	2.38E−05
*CXCR2*	1.67	1.15	2.2	0.000252
*CD14*	1.65	1.23	2.08	5.88E−05
*LILRB4*	1.64	1.16	2.12	0.000163
*IL1R2*	1.64	1.08	2.19	0.000426
*LILRA5*	1.58	1.11	2.05	0.000164
*PLAUR*	1.55	0.963	2.13	0.000829

Log2 FC = Log2 fold change (POD1-vs-DOS).

Lower = Lower confidence limit (log2).

Upper = Upper confidence limit (log2).

### RNA-seq analyses in PAXgene blood RNA confirmed the presence of surgery-induced circulating gene signatures

**Figure 3:**
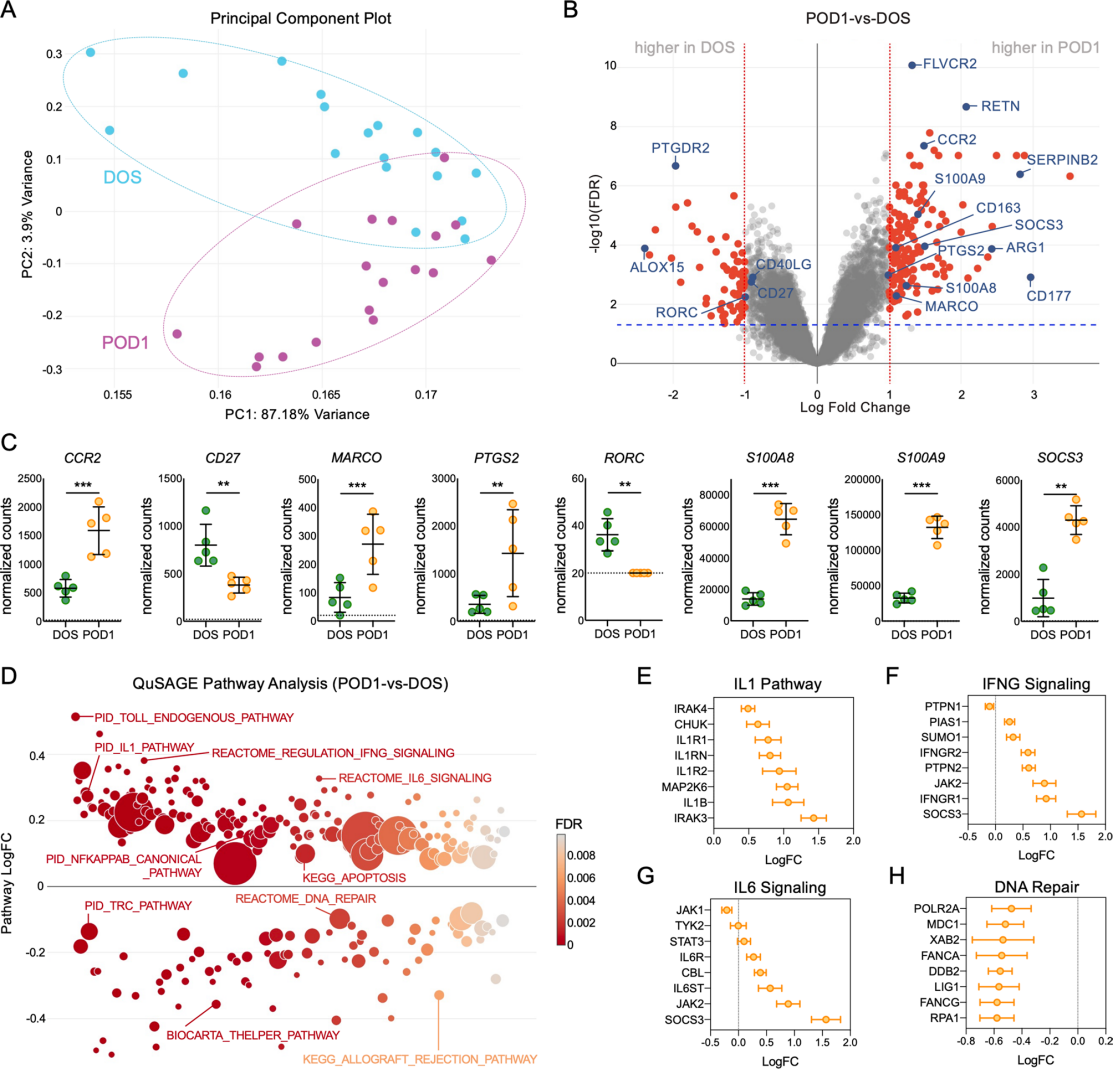
RNA-seq analyses of the responses to total knee arthroplasty in total RNA isolated from PAXgene blood RNA tubes. (**A**) Principal component analysis (PCA, using top 1,000 genes) of RNA-seq data from POD1 (24 h after surgery) and DOS (day of surgery). RNA-seq analysis is based on 36 samples obtained from 18 patients at DOS and POD1. (**B**) Volcano plot representing the differentially expressed genes comparing POD1 to DOS samples. Red dots correspond to genes with significant changes greater than 1 log2-fold expression change (FDR < 0.05, LogFC > 1). Selected genes with increased or decreased expression that were also identified by NanoString analyses are highlighted in blue. (**C**) NanoString normalized counts of selected DEGs, confirming changes identified by RNA-seq. Dotted lines indicate background signal. **p < 0.01, ***p < 0.001, by *t*-test, calculated using ratio data and the nSolver analysis software. NanoString data are represented as means ± S.D. (error bars) normalized counts. (**D**) Representation of the QuSAGE pathway analyses in POD1 relative to DOS, showing functional pathways differentially expressed at POD1 (FDR < 0.01). Representative genes associated with the (**E**) IL1, (**F**) IFNG signaling, (**G**) IL6 signaling, and (**H**) DNA repair pathways are shown, representing changes in gene expression (logFC) in POD1 vs. DOS samples. Graphs were created with GraphPad Prism 8 (GraphPad Software, San Diego, CA).

We next obtained high-quality RNA from PAXgene blood RNA tubes collected at DOS and POD1 from 8 patients who developed stiffness (cases, range of motion ≤ 95°) and 10 matched controls. RNA-seq analyses were first performed on PAXgene blood RNA from POD1 vs. DOS samples in all 18 patients to confirm the presence of surgery-induced signatures. These analyses uncovered unique transcriptional profiles in POD1 samples relative to DOS, as shown by the principal component analyses (PCA, Fig. 3A) and the volcano plot representation of DEGs (FDR < 0.05, LogFC > 1) between POD1 and DOS samples (Fig. 3B). See Supplementary Table S3 for a summary of all DEGs. Comparison of the RNA-seq (PAXgene blood) and NanoString (PBMCs) datasets uncovered similar DEG profiles at POD1, including changes in CCR2, CD27, MARCO, PTGS2, RORC, S10018, S100A9, or SOCS3 (Fig. 3C). Together, these changes in gene expression indicate that the PAXgene RNA-seq data provide a valid screening tool to identify surgery-induced changes. In spite of the strong similarities between datasets, these comparisons also uncovered differences between the DEGs identified in PBMCs and the RNA-seq analyses in PAXgene blood RNA (Supplementary Figure S2), which can be driven by the different platforms used for analyses, the differential presence of polymorphonuclear leukocytes (PMNs), or the osmotic stress associated with Ficoll purification, but are also consistent with reports highlighting differences between RNA from purified PBMCs and PAXgene blood RNA^28,29^.

Next, we performed QuSAGE pathway analyses^22^ using RNA-seq data (Fig. 3D, FDR < 0.01). Comparison of POD1 relative to DOS samples uncovered changes in pathways associated with inflammatory and reparative responses, including increased expression of the IL1 (Fig. 3E), IFNG (Fig. 3F), and IL6 signaling (Fig. 3G), and decreased expression of the DNA repair pathway at POD1 (Fig. 3H). See Supplementary Table S4 for a summary of all differentially expressed pathways in POD1 vs. DOS samples. Taken together, our RNA-seq analyses in PAXgene samples confirmed the presence of surgery-induced signatures early after TKA, detectable in whole blood and characterized by an overall upregulation of inflammatory and stress responses consistent with our NanoString gene expression data in PBMCs.

### Patients with knee stiffness at 6 weeks after TKA display distinct gene signatures at 24 h after surgery

Comparing cases and controls, we did not find significant differences for age (*p* = 0.95 by *t*-test), BMI (*p* = 0.32 by *t*-test), baseline flexion (*p* = 0.07 by *t*-test), baseline extension (*p* = 0.76 by *t*-test), baseline range-of-motion (*p* = 0.09 by *t*-test), and sex distribution (*p* = 0.19 by Fisher’s exact test). As expected, cases and controls had significant differences in post-operative range-of-motion (*p* = 0.0002 by *t*-test). Supplementary Table S5 summarizes the categorical demographics and the pre- and post-operative variables of cases and controls used for RNA-seq. All implants in the cases and controls included in this pilot study were cemented, with implant manufacturers similarly distributed between cases and controls. The same surgical approach was used for all patients (midline incision with a medial peripatellar arthrotomy). Further, we did not observe differences in tourniquet time between cases and controls (*p* = 0.94 by *t*-test).

**Figure 4:**
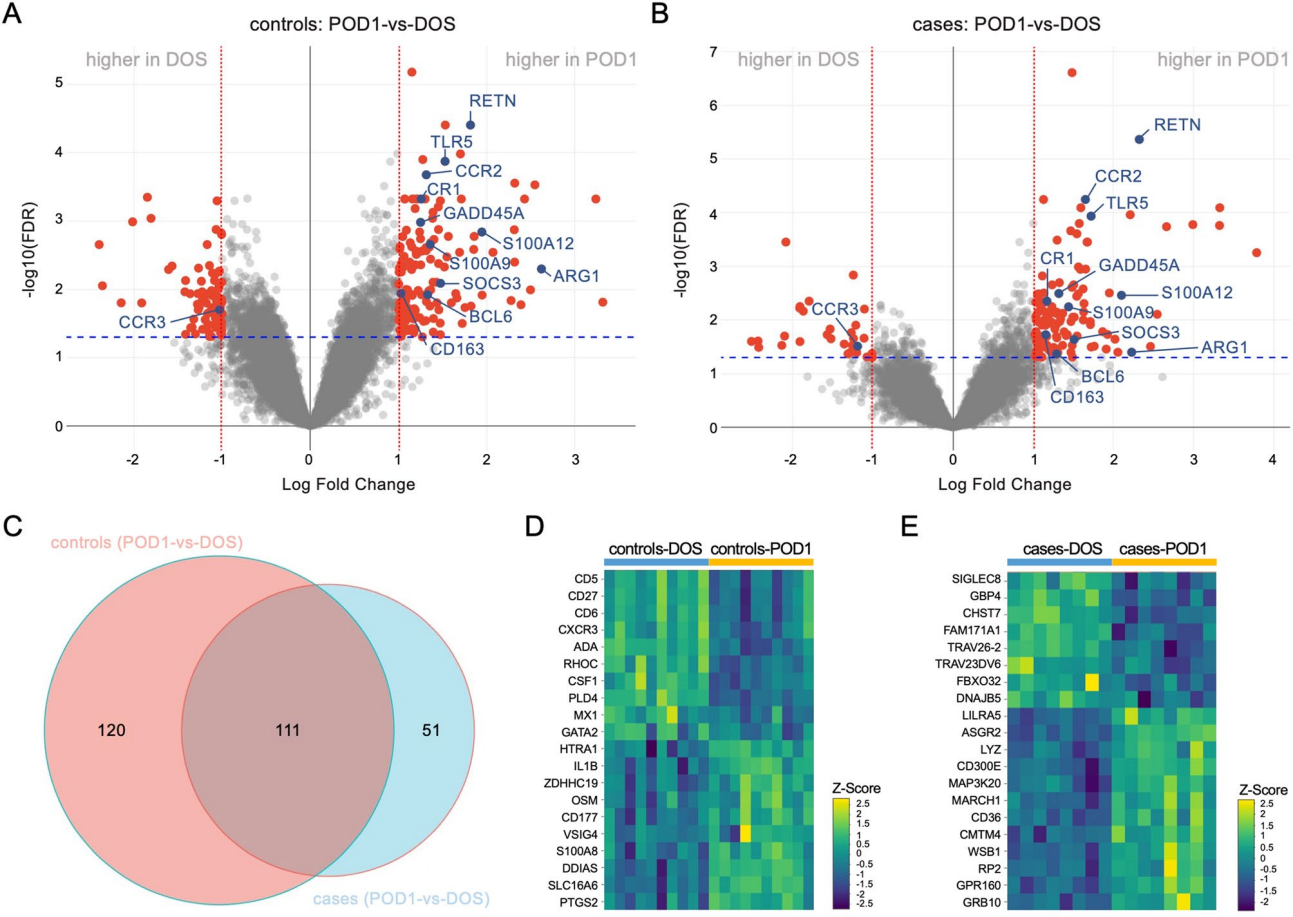
Transcriptomics analyses of PAXgene blood RNA identifies different gene signatures in cases (stiff knees) and controls following total knee arthroplasty. Volcano plots representing differentially expressed genes at 24 h after surgery (POD1) relative to the day of surgery (DOS) in (**A**) controls (no stiffness, N = 10) and (**B**) cases (stiff knees, N = 8). Red dots correspond to genes with significant changes greater than 1 log2-fold expression change (FDR < 0.05, LogFC > 1). Selected DEGs common to cases and controls are highlighted in blue. (**C**) Venn diagram representation of the DEGs at POD1 compared to DOS that are common to cases and controls (N = 111) or unique to controls (N = 120) and cases (N = 51). Heatmap representation of selected DEGs that are unique to (**D**) controls and (**E**) cases is shown. Data were z-score normalized.

We further analyzed our PAXgene RNA-seq dataset to identify perioperative gene signatures associated with stiffness at 6 weeks following TKA. We did not detect significant differences in gene expression comparing cases and controls at baseline, in DOS samples. Relative to DOS levels, we found different transcriptional profiles at POD1 in cases versus controls, as shown in the volcano plot representation of the DEGs (FDR < 0.05, LogFC > 1) for each group. Specifically, RNA-seq analyses uncovered 231 DEGs at POD1 in controls (Fig. 4A) and 162 DEGs in cases (Fig. 4B). The Venn diagram representation in Fig. 4C depicts the unique and overlapping genes in cases and controls. We identified 111 overlapping DEGs for cases and controls, including ARG1, CCR2, CCR3, RETN, S100A9, S100A12, and TLR5 (Fig. 4A–B), which represent a general response to TKA. Notably, the control group displayed 120 unique DEGs, including inflammatory mediators and damage-induced genes like IL1B, OSM, PTGS2, and S100A8 (Fig. 4D). The cases displayed 51 unique DEGs at POD1 relative to DOS, including CD36, GBP4, LYZ and MARCH1 (Fig. 4E). Thus, comparison of cases versus controls uncovered attenuated and more variable responses to surgery in cases, with 231 DEGs at POD1 in controls versus the 162 DEGs identified in cases. See Supplementary Table S6 for a summary of unique and overlapping genes in cases and controls.

**Figure 5:**
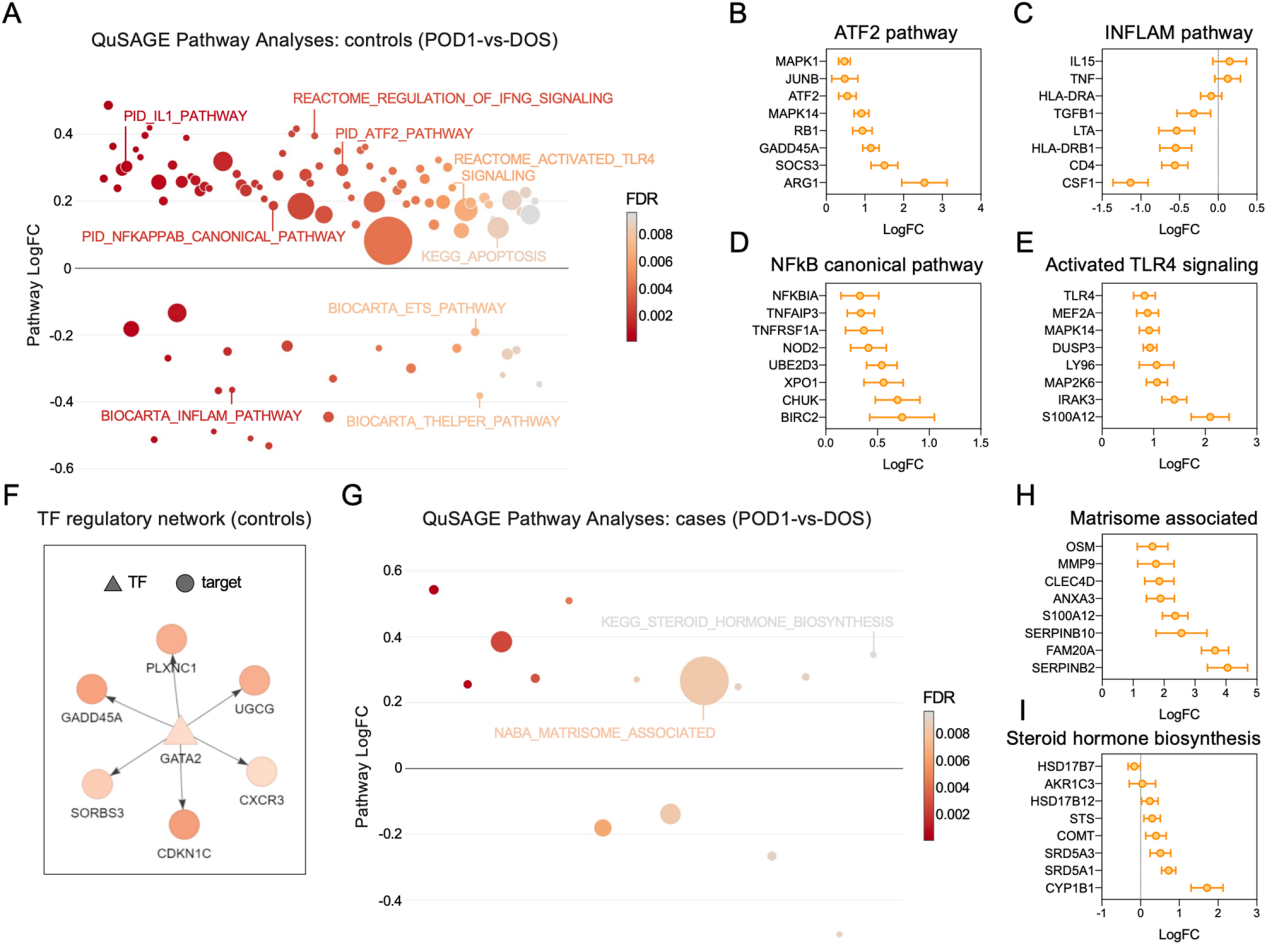
Functional analyses of the differentially expressed genes identified by RNA-seq in PAXgene blood RNA in cases (stiff knees) and controls. (**A**) Representation of the QuSAGE pathway analyses at 24 h after surgery (POD1) relative to the day of surgery (DOS) in control patients (N = 10) showing functional pathways differentially expressed at POD1 vs. DOS (FDR < 0.01). Representative genes associated with the (**B**) ATF2, (**C**) INFLAM, (**D**) canonical NF-κB signaling, and (**E**) activated TLR4 signaling pathways are shown, representing changes in gene expression (logFC) in POD1 vs. DOS for the controls. (**F**) Transcription factor regulatory network analysis using differentially expressed genes (logFC > 1, FDR < 0.05) at POD1 compared to DOS in the control group. (**G**) Representation of the QuSAGE pathway analyses in POD1 relative to DOS in cases (N = 8). Pathways that displayed differential expression (FDR < 0.01) only in the cases are highlighted. Representative genes associated with the (**H**) matrisome associated and (**I**) steroid hormone biosynthesis pathways are shown, representing changes in gene expression (logFC) in POD1 vs. DOS for the cases. Graphs were created with GraphPad Prism 8 (GraphPad Software, San Diego, CA).

QuSAGE pathway analyses^22^ using RNA-seq data also uncovered pronounced differences in cases and controls. Figure 5A summarizes the 97 differentially expressed signaling pathways identified in controls, comparing POD1 vs. DOS samples (FDR < 0.01), highlighting changes in the expression of the ATF2 pathway (Fig. 5B), pathways relevant to cytokines and inflammatory responses (Fig. 5C), the canonical NF-κB pathway (Fig. 5D), and the activated TLR4 signaling (Fig. 5E) after surgery. Similar to the network analyses using the combined POD1 and DOS samples (not shown), network analyses using the DEGs in the control group identified a transcription factor regulatory network involving GATA2, GADD45A, and CXCR3 (Fig. 5F). This transcriptional network was absent in the cases. Functional analyses using RNA-seq data from cases revealed a quite different transcriptional profile. QuSAGE analyses only uncovered 14 differentially expressed pathways in cases, comparing POD1 vs. DOS (FDR < 0.01, Fig. 5G). The majority of these pathways were common between cases and controls. The only 2 pathways unique to cases were the matrisome associated (Fig. 5H) and the steroid hormone biosynthesis (Fig. 5I). Important pathways involved in inflammatory, stress, and healing responses, including canonical NF-κB, p38 MAPK, signaling to ERKs, or the IL1 signaling pathway, were only differentially expressed after surgery in the control group. See Supplementary Table S7 for a summary of the QuSAGE pathway analyses in cases and controls.

Taken together, our RNA-seq analyses confirmed the presence of surgery-induced circulating gene signatures following TKA and showed that changes in these early gene signatures are associated with stiffness at 6 weeks after surgery.

## Discussion

In this pilot study, using comprehensive transcriptomics analyses in PBMCs and PAXgene whole blood RNA, we described the early circulating responses to surgery in patients undergoing TKA for OA. Integrating these analyses with clinical outcomes from our well characterized, prospectively enrolled cohort, we showed that changes in surgery-induced whole blood gene expression signatures associated with stiffness at 6 weeks after TKA can be detected early after surgery. Comparison of cases (patients who develop stiffness) versus controls uncovered attenuated and more variable responses to surgery in cases, with 231 DEGs at POD1 in controls versus the 162 DEGs identified in cases.

General responses to surgical and accidental trauma are well described^30,31^. Three recent studies, including our own, used targeted approaches and association with surgical outcomes to show changes in cytokine profiles and gene expression following TKA^9,14,15^. In the current study, using transcriptomics analyses in purified PBCMs and whole blood, we showed that TKA surgery leads to pronounced transcriptional changes in peripheral blood within 24 h, consistent with the previously described responses to trauma^30–32^ and the changes described in patients recovering from total hip arthroplasty^13^. To address the known potential artifacts that can be introduced during sample collection, handling, storage, and processing of whole blood preservation systems^33^, we compared RNA-seq from PAXgene blood RNA with NanoString data from PBMCs collected from 6 consecutive patients. Integrating NanoString and RNA-seq datasets, we found that monocyte/macrophage gene signatures are enriched at 24 h after surgery, with a concomitant increase in the expression of alarmins (e.g., S100A8), pattern recognition receptor signatures (e.g., TLR4), and inflammatory cytokines (e.g., IL1B), and with decreased T-cell gene signatures.

Macrophages exist within a phenotypic spectrum ranging from the classical (so-called M1) pro-inflammatory to the alternative (M2) state of activation, associated with the resolution of inflammation and healing. The coordinated action of these cellular components is critical to mediate host responses to tissue damage^34^. Our data do not permit single-cell resolution, so we cannot identify the relative enrichment of specific monocyte/macrophage subsets or activation states; however, we can identify a robust increase in monocyte signatures following surgery, including markers of M2-like macrophages like ARG1, CD163, and MARCO^35,36^. Soon after injury, and in parallel to the recruitment of macrophages and increased inflammation, there is also a compensatory phase characterized by reduced circulating T cell subsets, in part driven by increased migration into the periphery^13,37^. Our NanoString and RNAseq datasets agree with these previous reports and also show reduced T-cell gene expression at 24 h after surgery. Thus, the rapid and coordinated changes in gene expression signatures that we observed at 24 h after TKA surgery are consistent with the well-described general responses to injury, which are essential to drive optimal reparative processes.

Knee stiffness after TKA is often associated with the development of a fibrotic reaction in the knee joint (arthrofibrosis), indicating that impaired reparative responses lead to pathological scar formation concomitant with decreased range of motion^38–41^. In our study, comparison of cases (patients with stiff knees at 6 weeks after TKA) and controls (no stiffness) at baseline did not uncover significant differences in gene expression prior to surgery. However, comparison of the response to surgery in cases versus controls revealed a more variable and attenuated response to surgery in patients with stiffness after TKA. Comparing transcriptional profiles at a single-gene expression level we identified a common set of genes differentially expressed in response to surgery in both cases and controls, as well as subsets of genes that are unique and differentially expressed in controls (GATA2, IL1B, OSM, S100A8, and PTGS2) or in cases (LYZ, MARCH1, CD36, and RP2) after surgery.

When we evaluated functional gene signatures, we found that signaling pathways associated with stress, inflammation, and wound healing (including IL1, ATF2, or canonical NF-κB) are differentially expressed immediately after surgery only in controls, consistent with the expected response to injury and the immediate attempts to repair tissue damage^32,42^. The cases, however, did not display significant changes in the expression of these relevant signaling pathways at 24 h after surgery. This observation suggests that knee stiffness at 6 weeks post-TKA may be associated with changes in surgery-induced gene expression signatures that contribute to normal healing responses. Indeed, canonical NF-κB activation is observed in response to injury^43^, and the orchestration of canonical NF-κB targets is required for optimal tissue healing and repair and resolution of inflammation^44^. Thus, increased inflammatory and NF-κB-related responses in our control group can be interpreted as optimal reparative responses to trauma. However, we cannot conclude that the lack of changes in these pathways is proximate cause of stiffness in our cases. We did not evaluate whether the circulating/systemic responses mirror the inflammatory changes in the joint microenvironment that have been associated with fibrosis and stiffness^38,40^, and if the duration of these responses (or lack thereof) impacts outcomes, as is the case with persistent NF-κB activation leading to fibrotic healing^45^. Thus, the reproducibility, potential functional implications, and mechanistic relevance of our data in this context merit further investigation in larger and more diverse patient populations. Albeit not significant (p = 0.09 by *t*-test) the cases selected for this pilot study showed a relative enrichment in pre-operative stiffness (75% cases and 40% controls). While we did not observe differences in gene expression between cases and controls at baseline, we cannot rule out that (albeit not significant) the relative enrichment in pre-operative stiffness contributes to the attenuated gene expression observed in the cases after surgery. It is possible that the stimuli that lead to pre-operative stiffness modified the responses to surgery, akin to mechanisms whereby an initial exposure modify subsequent immune responses^46,47^. This should be evaluated in future work, comparing local and circulating signatures before and after surgery in patients without and with stiffness pre-operatively.

While the responses to surgery and the differences between cases and controls are robust and were obtained in a well-characterized patient cohort, our pilot study is not without limitations. Our data are correlative and do not allow us to establish direct mechanistic connections between peripheral gene expression and the development of stiffness and fibrosis after TKA, which has been shown to be associated with local inflammatory responses^38,40^. Our small sample size and the relative homogeneity of our patient population are limitations, and our data need to be validated and reproduced in larger and more heterogeneous cohorts. All patients enrolled in this study followed a standardized rehabilitation prototocol and we did not observe significant differences in baseline range-of-motion between cases and controls (*p* = 0.09). However, we did find that cases had higher pain Numeric Rating Scale (NRS) scores with movement prior to surgery (*p* = 0.02) despite no differences in baseline gene expression profiles. Preoperative knee pain predicts development of persistent pain following TKA^15,48–51^; however, the potential relationships between pain and other potential confounding and predisposing factors to knee stiffness, and their interaction with surgery-induced circulating responses, should be further analyzed in future studies and larger patient cohorts. The use of early stiffness (6 weeks) to identify cases and controls limits our ability to extrapolate our findings to patients who develop persistent or refractory knee stiffness and fibrosis requiring revision surgeries. However, 6 weeks after TKA is a clinically relevant time-point for determining the need for manipulation under anesthesia (MUA) as a means of recovering motion at the knee^11,52,53^. Indeed, 62% of the cases in this pilot study (5 out of 8 patients who developed stiffness at 6 weeks after TKA) had stiffness requiring MUA.

Finally, although PAXgene blood RNA tubes are a well-accepted and useful method for RNA collection and preservation in a clinical setting, RNA isolation from these samples is subject to technical and processing artifacts. These limitations, along with the small sample size used for RNA-seq and NanoString analyses, could have affected our ability to detect differences in cytokine transcripts with low expression levels and to establish comparisons with the protein data that we previously reported^9^.

In this pilot study, the changes in gene expression associated with stiffness after TKA were obtained in samples collected within 24 h after surgery. Future studies should aim to integrate these perioperative signatures with changes in gene expression detected at later time-points to identity signatures associated with the development of refractory knee stiffness. Future work should also aim to comprehensively address the predisposing factors leading to the development of stiffness and fibrosis, the association of stiffness with abnormal local and circulating immune responses, and the patient-intrinsic factors that contribute to the variable responses to treatment. These future studies should establish clinically relevant correlations between gene expression signatures and patient outcomes, aiming to identify patients and risk and prevent suboptimal outcomes. Additional studies that integrate clinical, histological, and multimodal cellular and genomics analyses on tissues retrieved at the time of primary TKA and revision surgeries can provide information about the pathways that, when dysregulated, contribute to poor recovery from TKA and the development of knee stiffness after surgery.

In conclusion, our results show that peripheral gene signatures can be used to evaluate pathways involved in the responses to surgery and that patients with stiffness following TKA may have dysregulated gene signatures detectable in the acute postoperative period. Notably, these gene expression signatures were detected from whole blood samples collected in PAXgene blood RNA tubes for transcriptomic analyses, suggesting a clinically feasible approach to developing molecular methods for predicting patients at risk of developing complications following surgery.

## Supplementary Information

Supplementary Information 1.

Supplementary Information 2.

Supplementary Information 3.

Supplementary Information 4.

Supplementary Information 5.

Supplementary Information 6.

Supplementary Information 7.

## Data Availability

The data that support the findings of this study are available from the corresponding author upon reasonable request. The RNA-seq sequencing data have been deposited at the database of Genotypes and Phenotypes (dbGaP) under dbGaP accession code phs002927.v1.p1.
