# Supplementary material for: Association of circulating gene expression signatures with stiffness following total knee arthroplasty for osteoarthritis: a pilot study
Source: Sci Rep. 2022 Jul 25;12:12651. doi: 10.1038/s41598-022-16868-y (PMC9314445; doi:10.1038/s41598-022-16868-y)
Supplement: Supplementary file 1 — Supplementary Information 1. [file 41598_2022_16868_MOESM1_ESM.pdf]

Supplementary Materials for

Association of circulating gene expression signatures with stiffness following total knee arthroplasty for osteoarthritis: a pilot study

Meghan A. Kirksey[1,2,3], Samantha G. Lessard[1,4,5†], Marjan Khan[1,4,5†], George A. Birch[1,2], David Oliver[1,4,6], Purva Singh[1,4,5], Valeria Rotundo[1,2], Alexandra Sideris[1,2], TKAF Consortium[††], Alejandro Gonzalez Della Valle[1,3,7], Michael L. Parks[1,3,7], Peter K. Sculco[1,3,7], Miguel Otero[1,3,4,5,6*]

Affiliations: [1]Hospital for Special Surgery, New York, NY, 10021, [2]Department of Anesthesiology, Critical Care, and Pain Management, Hospital for Special Surgery, New York, NY, 10021, [3]Weill Cornell Medical College, New York, NY, 10021, USA. [4]HSS Research Institute, Hospital for Special Surgery, New York, NY, 10021, USA. [5]Orthopedic Soft Tissue Research Program, Hospital for Special Surgery, New York, NY, 10021, USA. [6]The David Z. Rosensweig Genomics Research Center, Hospital for Special Surgery, New York, NY, 10021, USA. [7]The Stavros Niarchos Foundation Complex Joint Reconstruction Center, Hospital for Special Surgery, New York, NY, 10021, USA.

[†]Samantha Lessard and Marjan Khan contributed equally

[††]**TKAF CONSORTIUM:** Tania Pannellini[1,3], Allina A. Nocon[1,7], Mark Youseff[1,7], Paul Guirguis[1,7], Thomas W. Bauer[1,3], Eric A Bogner[1,3], Mathias P. Bostrom[1,3,4,7], Steven B. Haas[1,3,7], Kethy M. Jules-Elysee[1,2,3], Mark P. Figgie[1,3,7], David J. Mayman[1,3,7], Alexander S. McLawhorn[1,3,7], Michael B. Cross[1,3,7], Douglas E. Padgett[1,3,7], Alessandra B. Pernis[1,3,4,6], Scott A. Rodeo[1,3,4,5], Kathleen Tam[1,7], Geoffrey H. Westrich[1,3,7], Hollis G. Potter[1,3], Matthew F. Koff[1,3], Lionel B. Ivashkiv[1,3,4,6], Thomas P. Sculco[1,3,7], Timothy M Wright[1,3]

[1]Hospital for Special Surgery, New York, NY, 10021, [2]Department of Anesthesiology, Critical Care, and Pain Management, Hospital for Special Surgery, New York, NY, 10021, [3]Weill Cornell Medical College, New York, NY, 10021, USA. [4]HSS Research Institute, Hospital for Special Surgery, New York, NY, 10021, USA. [5]Orthopedic Soft Tissue Research Program, Hospital for Special Surgery, New York, NY, 10021, USA. [6]The David Z. Rosensweig Genomics Research Center, Hospital for Special Surgery, New York, NY, 10021, USA. [7]The Stavros Niarchos Foundation Complex Joint Reconstruction Center, Hospital for Special Surgery, New York, NY, 10021, USA.

*To whom correspondence should be addressed: Miguel Otero, Ph.D., Hospital for Special Surgery, HSS Research Institute, Orthopedic Soft Tissue Research Program, Room 603, 535 East 70th Street, New York, NY 10021, USA; Tel. 212-774-7561; Fax. 617-249-2373; E-mail: oterom@hss.edu

This file includes:

Figure S1: NanoString analyses of PBMCs obtained at DOS and POD1 (6 patients).
Figure S2: Comparison of RNA-seq in PAXgene blood RNA and NanoString in PBMCs.
Table S5: Demographics and pre- and post-operative characteristics of cases and controls used for RNA-seq analyses in PAXgene blood RNA.

Other Supplementary Materials for this manuscript include the following:

Table S1: Normalized counts of the final NanoString analyses in PBMCs (5 patients).
Table S2: Cell and pathway scores, NanoString analyses in PBMCs
Table S3: RNA-seq, differentially expressed genes
Table S4: QuSAGE pathway analyses, POD1-vs-DOS (all samples)
Table S6: RNA-seq Venn diagram gene subsets, cases and controls
Table S7: QuSAGE pathway analyses, POD1-vs-DOS in cases and controls

SUPPLEMENTARY FIGURES

Supplementary Figure S1: ***NanoString analyses in peripheral blood mononuclear cells (PBMCs) obtained the day of surgery (DOS) and at 24 hours after surgery (POD1) in patients undergoing total knee arthroplasty (TKA) for osteoarthritis (OA).*** Heat map representation of differential gene expression in RNA isolated from PBCMs from 6 knee OA patients undergoing TKA. Each column represents data from samples collected from one patient (see legend for samples corresponding to each patient, subjects #4 to #9) on the DOS (green) or at POD1 (gold). Each row represents the relative expression of different genes, with yellow indicating higher expression, and blue/black indicating lower expression. Data were Z-score normalized. Subject # 5 (red label) showed a different gene expression response to surgery relative to the other patients. The patient had received twice the dose of post-op ketorolac and meloxicam compared to the other patients, and samples from subject #5 were therefore not included in the final NanoString analyses.

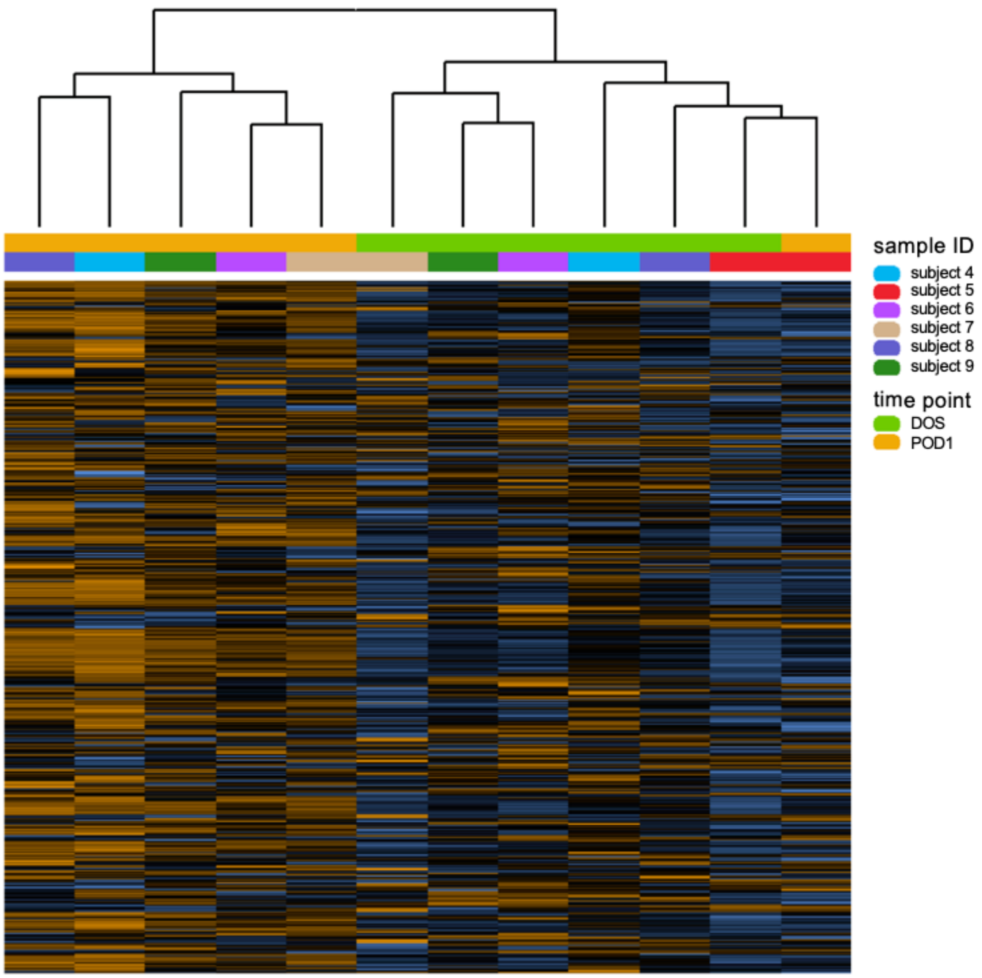

Supplementary Figure S2: ***Comparison of the RNA-seq (PAXgene blood RNA) and NanoString (peripheral blood mononuclear cells, PBMCs) datasets.*** **(A)** Normalized counts of selected differentially expressed genes (DEGs) identified by NanoString analyses in RNA from PBMCs isolated the day of surgery (DOS) and 24 h after surgery (POD1) from 5 patients undergoing total knee arthroplasty (TKA) for knee osteoarthritis (OA). Dotted lines indicate background signal. **p<0.01, ***p<0.001, ****p<0.0001 by *t*-test, calculated using ratio data and the nSolver analysis software. Graphs were created with GraphPad Prism 8 (GraphPad Software, San Diego, CA). (**B**) Volcano plot representation of the differentially expressed genes (red) at POD1 relative to DOS identified by RNA-seq analyses of PAXgene blood RNA tubes, collected from 18 patients undergoing TKA for knee OA. Genes highlighted in blue correspond to the selected DEGs identified by NanoString analyses in PBMCs, which do not show significant changes in expression at POD1 versus DOS in RNA-seq analyses of PAXgene blood RNA.

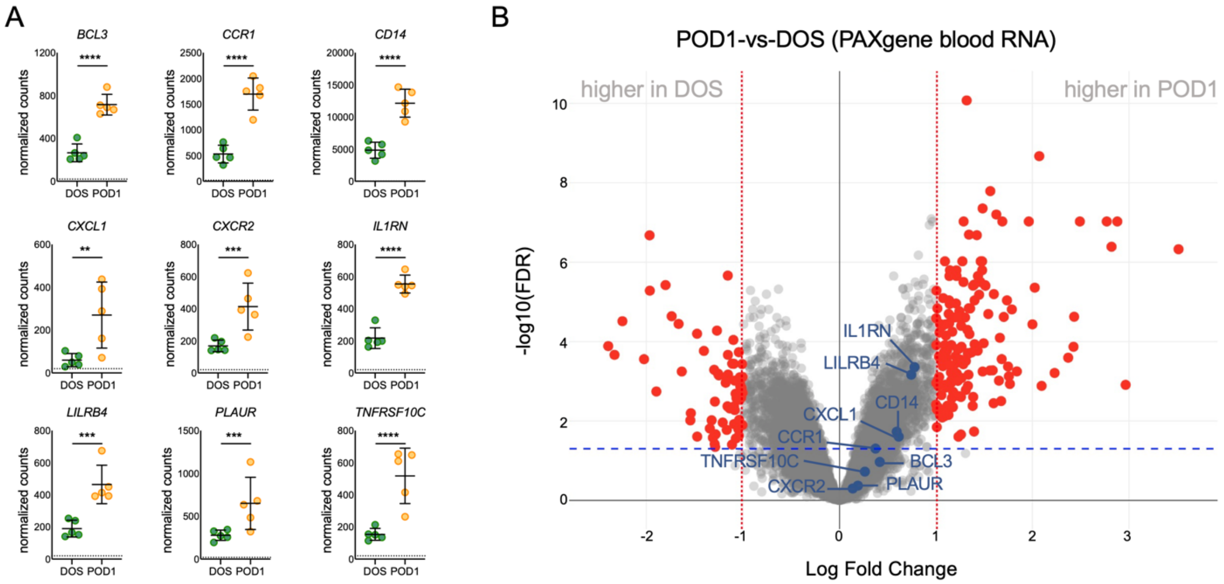

Supplementary Table S5: ***Demographics and pre-operative and post-operative characteristics of cases and controls used for RNA-seq in PAXgene blood RNA.***

	cases (n=8)	controls (n=10)	p value (t-test)
**age (years, mean ± s.d.)**	62.3 ± 6.255	62.80 ± 5.978	0.9525
**sex (male:female)**	2:6	6:4	0.189[a]
**BMI (kg/m2, mean ± s.d.)**	31.16 ± 8.621	35.01 ± 7.135	0.3153
**ethnicity (white:black/african american:asian)**	5:2:1	9:1:0	0.353[a]
**baseline flexion (degrees, mean ± s.d.)**	92.50 ± 30.00	112.5 ± 11.61	0.0695
**baseline extension (degrees, mean ± s.d.)**	6.250 ± 5.175	5.500 ± 4.972	0.7588
**baseline range-of-motion (degrees, mean ± s.d.)**	86.25 ± 32.38	107.0 ± 14.38	0.0868
**post-op flexion (degrees, mean ± s.d.)**	78.13 ± 22.51	115.5 ± 4.378	**<0.0001**
**post-op extension (degrees, mean ± s.d.)**	4.000 ± 4.243	1.800 ± 3.360	0.2366
**post-op range-of-motion (degrees, mean ± s.d.)**	74.13 ± 26.03	113.7 ± 5.498	**0.0002**

[a]Fisher's exact test
